# Comprehensive Investigation into the Impact of Degradation of Recycled Polyethylene and Recycled Polypropylene on the Thermo-Mechanical Characteristics and Thermal Stability of Blends

**DOI:** 10.3390/molecules29184499

**Published:** 2024-09-22

**Authors:** Wencai Zhang, Jun Shen, Xiaogang Guo, Ke Wang, Jun Jia, Junting Zhao, Jinshuai Zhang

**Affiliations:** 1 School of Chemistry and Chemical Engineering, Taiyuan University of Technology, Taiyuan 030024, China; zhangwencai@sxgkd.edu.cn (W.Z.); shenjun@tyut.edu.cn (J.S.); 2023521061@link.tyut.edu.cn (K.W.); 2 School of Traffic Engineering, Shanxi Vocational University of Engineering Science and Technology, Jinzhong 030619, China; jiajun@sxgkd.edu.cn (J.J.); zhaojunting@sxgkd.edu.cn (J.Z.); zhangjinshuai@sxgkd.edu.cn (J.Z.); 3 Thomas D. Larson Pennsylvania Transportation Institute, College of Engineering, Pennsylvania State University, University Park, PA 16802, USA

**Keywords:** recycled polyethylene, recycled polypropylene, molecular simulation, properties, mechanism

## Abstract

The impact of degradation on plastics is a critical factor influencing their properties and behavior, particularly evident in polyethylene (PE) and polypropylene (PP) and their blends. However, the effect of photoaging and thermal degradation, specifically within recycled polyethylene (rPE) and recycled polypropylene (rPP), on the thermo-mechanical and thermostability aspects of these blends remains unexplored. To address this gap, a range of materials, including virgin polyethylene (vPE), recycled polyethylene (rPE), virgin polypropylene (vPP), recycled polypropylene (rPP), and their blends with different ratios, were comprehensively investigated. Through a systematic assessment encompassing variables such as melting flow index (MFI), functional groups, mechanical traits, crystallization behavior, microscopic morphology, and thermostability, it was found that thermo-oxidative degradation generated hydroxyl and carboxyl functional groups in rPE and rPP. Optimal mechanical properties were achieved with a 6:4 mass ratio of rPE to rPP, as validated by FTIR spectroscopy and microscopic morphology. By establishing the chemical model, the changes in the system with an rPE–rPP ratio of 6:4 and 8:2 were monitored by the molecular simulation method. When the rPE–rPP ratio was 6:4, the system’s energy was lower, and the number of hydrogen bonds was higher, which also confirmed the above experimental results. Differential scanning calorimetry revealed an increased crystallization temperature in rPE, a reduced crystallization peak area in rPP, and a diminished crystallization capacity in rPE/rPP blends, with rPP exerting a pronounced influence. This study plays a pivotal role in enhancing recycling efficiency and reducing production costs for waste plastics, especially rPE and rPP—the primary components of plastic waste. By uncovering insights into the degradation effects and material behaviors, our research offers practical pathways for more sustainable waste management. This approach facilitates the optimal utilization of the respective performance characteristics of rPE and rPP, enabling the development of highly cost-effective rPE/rPP blend materials and promoting the efficient reuse of waste materials.

## 1. Introduction

Polyethylene (PE) and polypropylene (PP) stand as the most prolific plastic raw materials globally, contributing to over 70 million and 50 million tons, respectively [1]. Additionally, recent statistics indicate that recycled polyethylene (rPE) and recycled polypropylene (rPP) continue to dominate as the primary types of plastic waste, with significant quantities being generated [2]. In contrast to energy-intensive methods and landfill disposal, the strategy of reuse has emerged as a highly effective means of waste plastic management. This approach not only mitigates environmental pollution but also serves as a resource-conserving solution [3]. By emphasizing reuse, we not only address the challenges posed by plastic waste but also contribute to sustainable practices that align with environmental preservation and resource efficiency.

The impact of degradation on the properties of polyethylene (PE) and polypropylene (PP) holds paramount significance, supported by an extensive body of research [4,5]. A study conducted on recycled polyethylene (rPE) and recycled polypropylene (rPP) from landfill sites revealed noteworthy differences. After more than 10 years of burial, the carbonyl index of rPP was found to be 2~3 times higher than that of virgin PP (vPP), while the -CH_2_ and -CH_3_ content in rPE and rPP was lower than that in virgin PE (vPE) and vPP. Additionally, the crystallinity of rPE and rPP was 1.5 times higher than that of vPE and vPP [6].

The influence of environmental factors, such as UV radiation and oxygen, on photo-oxidation and degradation mechanisms indicated that PP is prone to generating free radicals at high temperatures. This is attributed to the high reactivity of hydrogen connected with tertiary carbon atoms in PP, accelerating the degradation and decomposition of the material, thereby affecting the performance of rPP [7,8]. Moreover, the degradation of PE and PP is affected by the direct participation of water molecules in oxidation reactions and the indirect change in oxygen permeability [9].

Pressure load also plays a role in the degradation performance of PE pipes. Tests revealed a significant reduction in the lifetime of pipes under cyclic pressure, approximately 27.96% shorter than that under constant pressure [6]. Sudesh S. Frenando et al. [10] explored the formation and change rules of CO_2_ during the thermal and photodegradation of PE and PP. The study found that PE produced CO_2_, while PP did not. This was attributed to the slow development of carboxyl groups, providing further insights into the degradation mechanism of PE and PP. In summary, the factors influencing the degradation performance of PE and PP encompass three aspects: the material properties, external environmental factors, and internal load. Despite their similar hydrocarbon makeup, rPE/rPP blends are thermodynamically immiscible due to the positive Gibbs free energy of the blending system [1,11]. These blends may undergo reactions involving active functional groups generated during degradation.

The blending system of rPE and rPP has garnered extensive attention due to its low cost, availability, processability, and complementary properties [12]. Various studies have explored the mechanical properties, with findings indicating differences between copolymers, such as recycled low-density polyethylene (rLDPE)/homogeneous rPP and rLDPE/block rPP [13]. Furthermore, blending recycled high-density polyethylene (rHDPE) and rLDPE with rPP showed variations in performance, with rHDPE/rPP outperforming rLDPE/rPP due to similar structures resulting in more interfacial entanglement [14].

Numerous studies have also focused on the compatibilization modification of rPE/rPP blends, employing methods such as non-reactive compatibilizers [15], reactive compatibilizers [16], optimal technical conditions, et al. [17,18]. However, it is essential that we consider the purity of rPE and rPP raw materials, diverse proportions, degradation processes, and blending modification technologies, as they collectively impact the properties of the materials post-blending modification, consequently influencing the performance of the resulting products [19].

In summary, while numerous studies have delved into the realm of rPE/rPP blends, the majority have focused on comparing related properties, leaving a gap in understanding the broader impact of overall degradation, encompassing both photoaging and thermal degradation, on blending properties. To address this research gap, we set out to investigate the mechanical and rheological changes observed in rPE and rPP, particularly concerning degradation, and explore how alterations in molecular chains and chemical structures, resulting from degradation, affect the material properties of rPE/rPP blends.

Our study aimed to conduct a comprehensive comparative analysis of key properties, including melt index, mechanical properties, and micromorphology, of vPE, rPE, vPP, and rPP. We sought to uncover the influence of varying proportions of rPE/rPP on the blend properties. To enhance the depth of our examination into how degradation impacts these blends, we incorporated evidence-based analyses. Notably, our findings revealed distinct changes in mechanical traits, such as tensile strength and impact resistance, in rPE and rPP due to degradation, supported by quantitative data from rigorous testing protocols. Moreover, Fourier-transform infrared spectroscopy (FTIR) analyses provided concrete evidence of alterations in chemical structures, corroborating the formation of new functional groups during degradation. Additionally, scanning electron microscopy (SEM) images presented a compelling visual representation of micro-morphological changes within the blends, further substantiating the influence of degradation on the material’s internal structure.

By integrating these evidential analyses, our research not only contributes to the existing body of knowledge on rPE/rPP blends but also provides a more nuanced understanding of the intricate interplay between degradation processes and the resulting properties of these recycled polymer blends.

## 2. Results and Discussion

### 2.1. The MFI Results of vPE, rPE, vPP, and rPP

**Figure 1:**
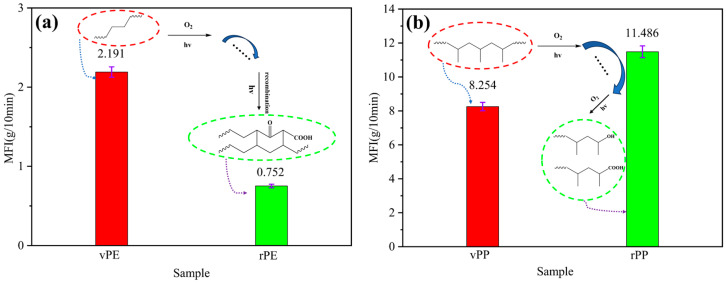
The MFI of vPE, rPE (**a**) and vPP, rPP (**b**).

The MFI of the polymer is affected by many factors and is closely related to its processing performance, which can reflect the relative molecular weight size and distribution law of polymer materials to a certain extent [20]. From Figure 1a, it can be observed that the MFI of vPE and rPE was 2.191 g/10 min and 0.752 g/10 min, respectively. Furthermore, the MFI decreased by 65.68% after degradation, because the vPE was subjected to physical and chemical effects during the service period, such as photo-oxygen degradation and thermal oxygen degradation, and it can be concluded that the degradation reaction mainly occurred in the early stage of degradation. During this period, the molecular chains and molecular weight decreased, making the movement of polymer segments relatively easier, which led to an increase in the MFI [21]. In the later stages of degradation, cross-linking reactions occurred, causing a sharp increase in molecular weight and a slowdown in polymer chain movement. This explains why the MFI initially increased and then decreased. This was consistent with the law discovered by Kartalis et al. [22] and Atiqah et al. [23]. In this study, the rPE might experience a longer degradation time so that the tested rPE was in the final stage, and the MFI decreased.

As shown in Figure 1b, it can be noted that the MFI of vPP and rPP was 8.254 g/10 min and 11.486 g/10 min, respectively. In contrast, the MFI of rPP that experienced natural degradation increased by 3.232 g/10 min (i.e., increasing 39.16%), which might be caused by the breaking of the molecular chain, a reduction in the molecular weight, and a decrease in viscosity under the combined effects of light, heat, and oxygen [24].

### 2.2. The MFI Results of vPE/vPP and rPE/rPP

**Figure 2:**
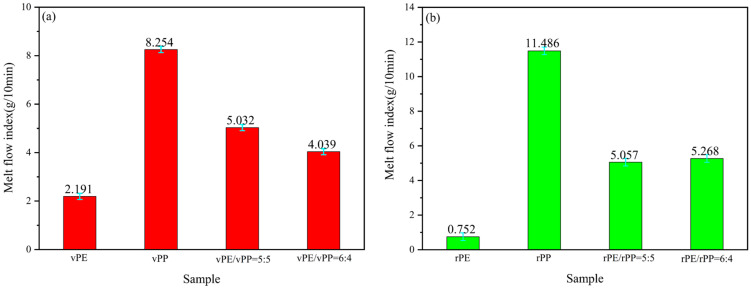
The melt flow index of vPE/vPP (**a**) and rPE/rPP (**b**).

Figure 2a showed the MFI results of a blend with vPE, vPP, and vPE/vPP ratios of 5:5 and 6:4, respectively. The MFI of the vPE/vPP mixture with a ratio of 5:5 was 5.032 g/10 min, and theoretical calculation was 5.223 g/10 min. The difference between the two values was very small, indicating the blending process was a physical mixture. In addition, the viscosity of the vPE/vPP mixture is slightly larger than the virgin samples, meaning there was part of the molecular chain entanglement behavior. As the vPE content increased, the MFI of the vPE/vPP mixture decreased. Therefore, when the ratio of vPE to vPP was 6:4, the MFI was 4.039 g/10 min, lower than the calculated value of 4.616 g/10 min indicating that the influence of vPE on the MFI of the blend was dominated by physical blending process. Additionally, from a statistical standpoint, the error between 5.032 and 5.223 is 4%, whereas the error between 4.616 and 4.039 is 15%, marking a significant disparity in magnitude. It was evident that a 4% error was generally acceptable, while a 15% error was unacceptable.

Figure 2b presents the MFI of the rPE, rPP, and rPE/rPP mixtures with 5:5 and 6:4 mass ratios, respectively. It can be seen that the MFI of the rPE/rPP blends with a 5:5 mass ratio was 5.057 g/10 min, while the calculated MFI of rPE and rPP was 6.119 g/10 min. There was an obvious difference between these two values, and the MFI of rPE/rPP blends was lower, indicating that the rPE and rPP have a certain degree of entanglement between molecular chains in addition to physical blending [25]. Due to the degradation of vPE and vPP, there were esterification reactions of the hydroxyl group and carboxyl group after rPE and rPP aged, resulting in an increase in the molecular chain and then leading to an increase in viscosity and decrease in MFI. As the ratio of rPE to rPP increased to 6:4, the MFI slightly increased to 5.268 g/10 min, indicating that the chemical reactions between rPE and rPP had a stronger effect on the viscosity by comparison in the influence of the rPE component.

Under identical test temperature conditions, rPE initially undergoes significant sub-chain breakage, leading to a reduction in molecular weight and increased mobility of polymer chain segments, which results in a relatively high melt index. However, in the later stages, the melt index decreases. As described in references [22,23], this decrease is attributed to the cross-linking effect, which restricts the movement of the polymer chains.

### 2.3. The Mechanical Properties of vPE, rPE, vPP, and rPP

Table 1 presents the mechanical properties of vPE, rPE, vPP, and rPP, showing that the tensile strength, elongation, notched impact strength, and bending strength of vPE and vPP deterioration after degradation. Among these properties, the variation of elongation of rPE and rPP was the most obvious and reduced by 73.74% and 37.16%, compared with their respective virgin materials.

For vPE, the deterioration mechanism of mechanical properties was structural variation during the age period. Prior to vPE degradation, the molecular chain was intact, and the structure was compact, contributing to excellent mechanical properties. In the early degradation stage, the surface molecular chain of rPE was oxidized and fractured under the interactions with light, heat, and oxygen. Thus, the molecular weight was reduced, and surface cracks appeared. With the extension of the degradation time, oxygen and heat penetrated the material and then led to the oxidation of the internal molecular chain. Due to the formation of oxygen-containing functional groups, the cross-linking interaction between molecules became the dominant reaction, which was consistent with the change in MFI. In conclusion, the diffusion of surface cracks and the further degradation of PE materials were the main causes of the decrease in comprehensive mechanical properties [26].

For vPP, the reason for the decline in mechanical properties was the vulnerable tertiary carbon atoms under light, heat, and oxygen attacks. By the comprehensive influences of three factors, the molecular chain of rPP was oxidized and broken. Subsequently, the molecular weight decreased, and surface cracks initially formed, gradually extending internally, ultimately leading to a decline in mechanical properties.

### 2.4. The Mechanical Properties of rPE/rPP

**Figure 3:**
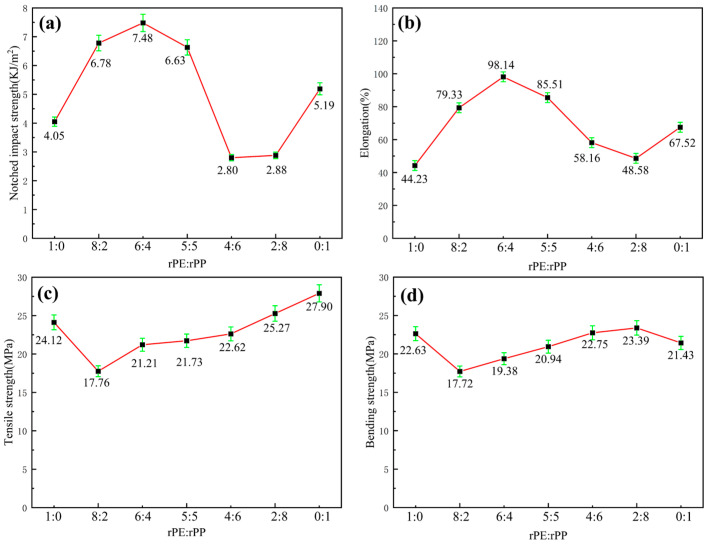
The mechanical properties of the rPE/rPP mixture: (**a**) notched impact strength, (**b**) elongation, (**c**) tensile strength, and (**d**) bending strength.

The rPE/rPP mixture was prepared with the mass ratio of 1:0, 8:2, 6:4, 5:5, 4:6, 2:8, and 0:1. As shown in Figure 3a, the notched impact strength of pure rPE was 4.05 kJ/m^2^. With the addition of rPP with a mass ratio of 8:2, the notched impact strength of the mixture increased to 6.78 kJ/m^2^. The notched impact strength of pure rPP was only 5.19 kJ/m^2^, indicating there was a certain degree of chemical reactions happened between rPE and rPP. The chain entanglement between rPE and rPP became tight and the notch impact strength increased. With the increase in rPP content, the notched impact strength further increased. When rPE/rPP mass ratio was 6:4, the notched impact strength reached a maximum value of 7.48 kJ/m^2^, meaning that the reactions between rPE and rPP reached the equilibrium. Excess hydroxyl and carboxyl groups increased system polarity, which was incompatible with the non-polar nature of the new material blend. Impact strength was the most crucial property, exhibiting significant differences among different blends, whereas tensile and bending strengths showed minimal variations. Therefore, impact strength served as the primary criterion. In other proportions, the presence of excessive hydroxyl and carboxyl groups would similarly increase system polarity, leading to reduced compatibility and balance within the system.

As the rPP content continuously increased, the notched impact strength gradually decreased, which might be caused by the excess of polar groups in rPP and declined compatibility, such as hydroxyl groups and carboxyl groups. When the rPE/rPP mass ratio was 4:6 or 2:8, the notched impact strength was the lowest, indicating that the polar group content in the mixture was the highest. It had been reported that the rPE/rPP mixture was prepared by a double-rotor mixer and the mechanical properties were related to rPE content [19]. The impact strength of the rPE/rPP mixture rapidly increased with the increase in rPE content, which was confirmed by the results of SEM, where the small filaments in the impact section appeared and the rPE played the role of flexibility. The reason for this was that the mechanical properties of rPE surpassed those of rPP, and there was a significant interaction between these materials. However, the precise reaction mechanism that affected the impact strength of the rPP/rPE mixture remained unclear.

Figure 3b showed the elongation at the break of the rPE/rPP mixture. It can be observed that the elongation at break of pure rPE and pure rPP was 44.23% and 67.52%, respectively. As the proportion of rPE increased, the elongation at the break of the blend increased. When the mass ratio of rPE/rPP was 6:4, the elongation at break reached a maximum of 98.14%, which is increased by 121.89% and 45.35% compared with pure rPE and pure rPP, respectively. With the rPP content dominating, the elongation at break of the rPE/rPP mixture decreased. The changes in elongation at break with varying ratios of rPE to rPP aligned closely with the notch impact strength, providing further confirmation of chemical reactions that occurred between rPE and rPP. These reactions ultimately led to an increase in toughness.

Figure 3c presented the tensile strength of the rPE/rPP mixture. It can be observed that the tensile strength of rPE and rPP was 24.12 MPa and 27.90 MPa, respectively. When 20% rPP was added to pure rPE, the tensile strength decreased sharply, which might be caused by the poor compatibility and weak interfacial bonding force between rPP and rPE. Conversely, the tensile strength increased with a higher rPP content due to the greater rigidity and strength inherent in rPP. In cases where the proportion of rPP was higher, the dominant rigidity of rPP led to an overall increase in the blend’s strength.

Figure 3d showed the bending strength of rPE/rPP mixtures. The variation curve was consistent with that of tensile strength. However, when the mass ratio of rPE to rPP was 2:8, the bending strength reached the highest point and exceeded the pure rPE and rPP materials, indicating that there was a certain compatibility between rPE and rPP, resulting in a further increase in flexural strength.

In summary, the rPE–rPP ratio has a minimal impact on the tensile strength and bending strength compared to the notched impact strength and elongation at break. Additionally, the mechanical properties analysis indicates that an rPE–rPP ratio of 6:4 provides superior toughness in the blend, demonstrating significant practical value. Therefore, we recommend an optimal mass ratio of rPE to rPP of 6:4.

### 2.5. The FTIR Analysis

**Figure 4:**
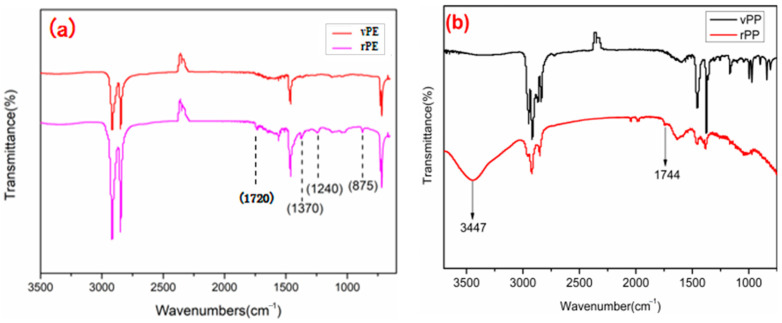
The infrared spectrogram of vPE and rPE (**a**), vPP and rPP (**b**).

Figure 4a showed the FTIR spectra of vPE and rPE. The peak of rPE at 1240 cm^−1^ was significantly enhanced compared to vPE, corresponding to the in-plane bending vibration peak of alcohol hydroxyl groups. A new peak was generated at 1720 cm^−1^, corresponding to the stretching vibration peak of the carbonyl group. At about 3300 cm^−1^ to 3500 cm^−1^, rPE had a weak broad peak, corresponding to the stretching vibration peak of the hydroxyl group in the carboxyl group [27], indicating that, during the use of vPE, under the combined action of oxygen, heat, and ultraviolet light, new hydroxyl, carbonyl, or carboxyl groups were generated. At 875 cm^−1^ and 1370 cm^−1^, the corresponding in-plane bending vibration peaks of methyl groups were observed [28]. The increase in peak strength indicated that molecular chain breakage caused an increase in the number of molecular chains relative to vPE, increasing the number of methyl groups. Based on the analysis, it was speculated that there were various factors affecting the degradation of PE, such as the branch chain number and crystallinity. The larger the branch chain number, the more the tertiary carbon, and the easier to age.

**Figure 5:**
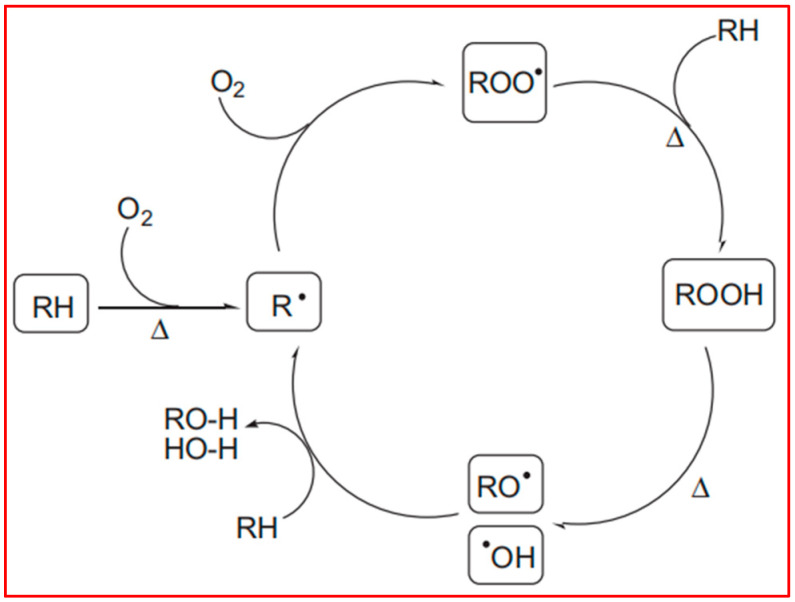
The mechanism of cycle thermal-oxidative degradation for PP.

Figure 4b was the FTIR spectra of vPP and rPP. It is evident that rPP exhibited a shoulder peak at 1744 cm^−1^, associated with the carbonyl peak, albeit with a relatively low intensity. This observation could be attributed to the short service life and limited degree of degradation in rPP, indicating it was in a relatively early stage of degradation, characterized by the emergence of hydroxyl groups. Within the range of approximately 3000 cm^−1^ and 3700 cm^−1^, rPP displayed a broad peak, corresponding to the stretching vibration peak of hydroxyl groups in the carboxyl group. This suggested that, during the use of vPP, under the combined influence of oxygen, heat, and ultraviolet light, new hydroxyl groups, as well as a small quantity of carbonyl or carboxyl groups, were generated. In this study, the degradation and thermal degradation process of vPP was shown in Figure 5, which was consistent with the mechanism agreed upon by Denis Bertin et al. [29]. Polyolefin polymers contain numerous tertiary carbon atoms along their long carbon chains. The C–H bonds at these tertiary carbon sites are relatively weak, rendering them particularly susceptible to degradation induced by external factors such as heat, oxygen, and light. Under these conditions, hydrogen atoms can be abstracted from the tertiary carbon atoms, leading to the formation of free radicals (R°), which, in turn, initiate processes such as fragmentation and cross-linking. The current understanding of the free radical degradation mechanism suggests several sequential steps: the generation of free radicals, hydrogen abstraction by these radicals, and the subsequent recombination or scission of polymer free radicals. Furthermore, in the presence of oxygen, free radicals can react to form peroxy radicals, which continue to attack tertiary carbon atoms, producing hydroperoxides. These newly formed alkyl radicals perpetuate the degradation process, resulting in the further breakdown of polyolefin molecules.

The photodegradation mechanism in polyolefins involves the excitation of light-sensitive functional groups, such as carbonyls, from their ground state to an excited state. This excitation occurs via atomic or electron transfer, producing active free radicals that initiate a cascade of chemical reactions, ultimately leading to chain scission. In subsequent reactions, the intermediates generated during these processes introduce functional groups such as carbonyls, hydroxyls, and vinyls onto the polyolefin backbone through mechanisms like O–O bond cleavage and hydrogen transfer, thus accelerating the degradation process.

The observed differences in the FTIR spectra between recycled polyethylene (rPE) and virgin polyethylene (vPE) can be attributed to the chemical changes that occur during the recycling process. The additional absorption peaks and increased intensities in the rPE spectrum are primarily due to oxidative degradation and the formation of new functional groups. During recycling, polyethylene undergoes thermal and oxidative stress, leading to the formation of carbonyl groups (observed at 1720 cm^−1^) and hydroxyl groups (observed at 1240 cm^−1^). These groups are products of oxidation reactions that degrade the polymer chains.

The increased intensity of peaks associated with methyl groups (at 875 cm^−1^ and 1370 cm^−1^) further supports the presence of degradation products and indicates significant changes in the polymer structure. These changes are intrinsic to the degradation mechanisms affecting rPE and reflect the impact of the recycling process on the material’s chemical composition [28].

**Figure 6:**
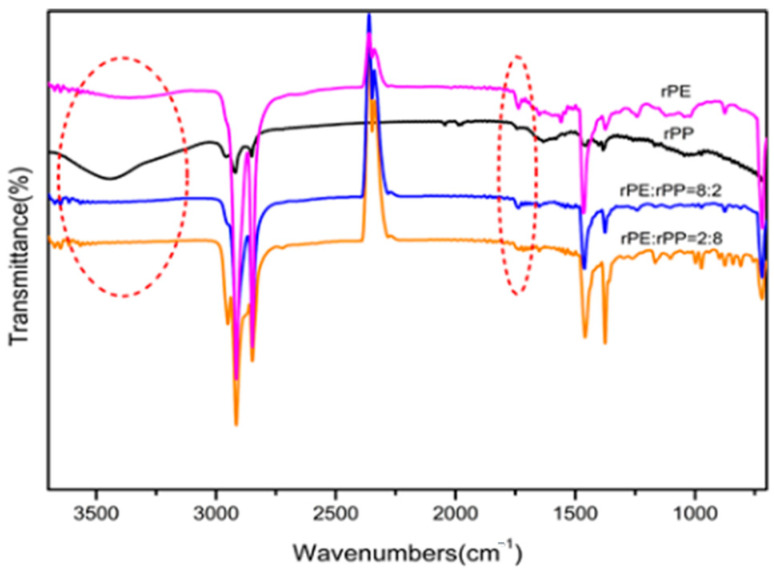
The infrared spectrogram rPE, rPP, and rPE/rPP blends.

Figure 6 was the FTIR spectra of the rPE, rPP, and rPE/rPP blends. It can be seen that the characteristic peaks at 3000 cm^−1^ to 3500 cm^−1^, corresponding to the stretching vibration peaks of hydroxyl groups in the carboxyl group, disappeared after blending rPE with rPP. However, there was no significant change in the peak of 1740 cm^−1^, which corresponded to a carbonyl peak, indicating that there was a certain degree of esterification reaction between the hydroxyl and carboxyl groups generated by degradation in rPE and rPP, resulting in a significant decrease in the number of carboxyl groups. The presence of a carbonyl peak around 1740 cm^−1^ in the ester group, without disappearance, provided further evidence of an esterification reaction occurred after the blending of rPE and rPP.

The chemical interactions between rPE and rPP during blending are primarily driven by esterification reactions as shown in Figure 6. These reactions occur due to the presence of reactive functional groups—carboxyl and hydroxyl groups—that are generated in the polymers as a result of their prior aging. The extrusion process, involving high temperatures, shear forces, and mechanical mixing, provides the necessary conditions for these groups to interact and form ester bonds.

The esterification reaction is supported by the infrared spectra. The spectra of the rPE/rPP blends show new absorption peaks corresponding to ester groups, which are not present in the spectra of the individual polymers. This indicates that esterification has occurred during the blending process. The reaction enhances the compatibility between rPE and rPP, leading to improved properties of the recycled blends compared to those made from virgin materials.

### 2.6. Molecular Dynamics Simulation Analysis

**Figure 7:**
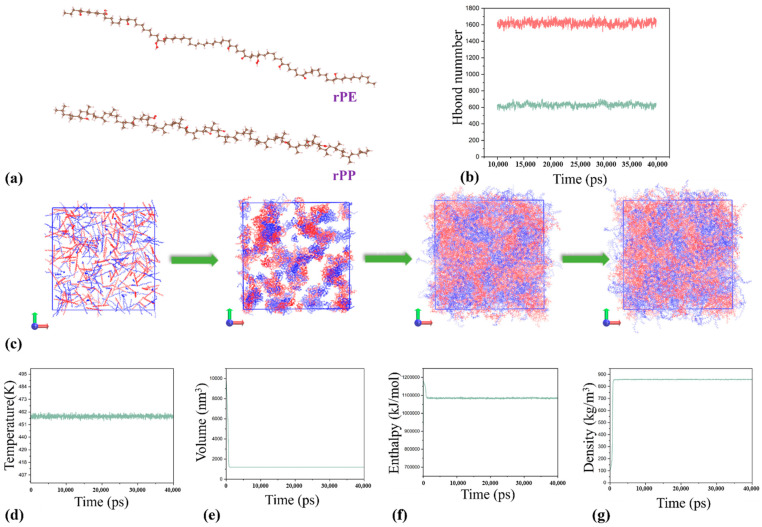
Molecular structure diagram of rPP and rPE (**a**). Number of hydrogen bonds during molecular simulation (**b**). Molecular simulation process (**c**). Temperature (**d**). Volume (**e**). Enthalpy (**f**). Density (**g**).

The extended exposure of discarded rPP and rPE to the environment led to oxidation reactions. Therefore, to model these molecular systems, oxidation groups were incorporated into the rPP and rPE chain segments, as depicted in Figure 7a. The oxidation groups incorporated into the rPP and rPE chain segments include hydroxyl and carbonyl groups. Specifically, each polymer chain segment contains an average of five hydroxyl groups and three carbonyl groups. These functional groups were introduced to modify the properties of the polymers. Subsequently, simulations were conducted under the NPT ensemble using the GAFF force field. The simulation employed a time step of 2 fs and ran for a total runtime of 40 ns. Temperature and pressure were controlled using the V-rescale and C-rescale during the MD simulations. The van der Waals interaction cutoff distance was set to 12.5 Å. The simulation process of the rPE/rPP blend was illustrated in Figure 7c, with a composition ratio of 4:6. The Figure 7c demonstrated a high degree of compatibility between the two components. Throughout the NPT simulation, parameters including temperature, volume, energy, and density were monitored to ensure simulation convergence, as depicted in Figure 7d–g. Additionally, Figure 7f indicated a reduction in system energy post the blending of rPE/rPP, suggesting a compatibility between rPP and rPE. In Figure 7b, the red solid line represented the total number of hydrogen bonds within the system, while the green solid line denoted the number of hydrogen bonds formed between rPP and rPE. This observation underscored the significant hydrogen bonding between rPP and rPE, contributing to their favorable compatibility [30,31]. The molecular motion process was recorded in a video. Please refer to Supplementary Materials, the video titled ’MD Simulation Trajectory’.

**Figure 8:**
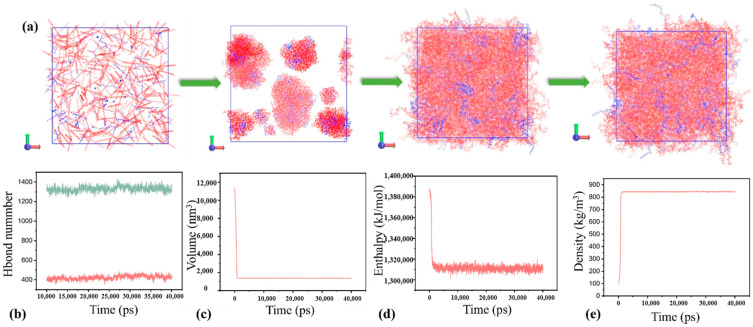
Molecular simulation process (**a**). Number of hydrogen bonds during molecular simulation (**b**). Volume (**c**). Enthalpy (**d**). Density (**e**).

Using the same simulation methods and approach as described above, the blended rPE/rPP model with a ratio of 2:8 was constructed, as illustrated in Figure 8a. It is evident that, even with the passing of time, the two components continue to demonstrate a significant level of compatibility. Notably, the enthalpy of the system in Figure 8d exceeded that in Figure 7f, suggesting that the 2:8 rPE–rPP ratio system was less stable compared to the 4:6 ratio system. This observation was corroborated by the hydrogen bond analysis, where the number of hydrogen bonds between rPE and rPP in Figure 8b was one-third fewer than in Figure 7b. The molecular simulation results highlighted that rPE and rPP exhibited robust compatibility at a 4:6 ratio, primarily due to the substantial presence of hydrogen bonds between them. Conversely, altering the ratio to 2:8 resulted in an increased system enthalpy and a reduced hydrogen bond count, indicating diminished compatibility. These findings aligned consistently with the aforementioned experimental outcomes and existing research [32].

### 2.7. The Thermal Performance Analysis

**Figure 9:**
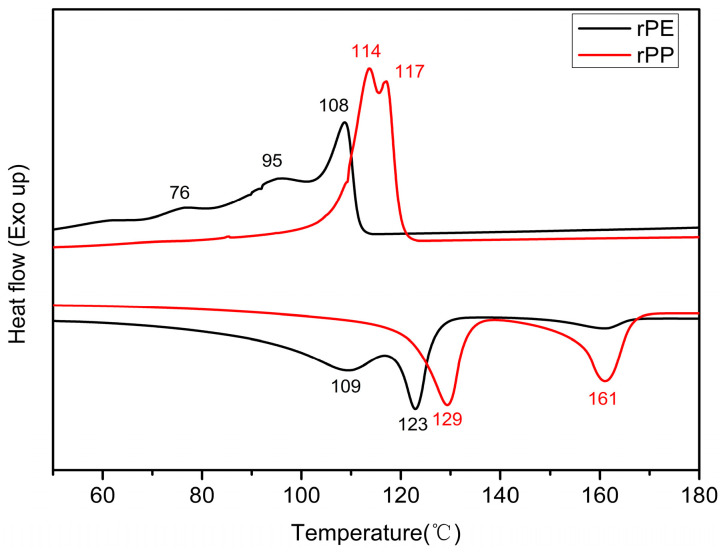
The DSC curves of rPE and rPP.

Figure 9 showed the DSC curves of rPE and rPP. The crystallization temperature of rPE was around 108 °C, and the shoulder peak near 95 °C might be a region peak with a relatively low degree of PE crystallization perfection. On one hand, it might be that the composition of rPE contains multiple structures, such as HDPE and LDPE, so shoulder peaks appeared near 95 °C and 76 °C. On the other hand, it might be because rPE had a decreased molecular weight due to degradation, resulting in molecular chain breakage, and causing damage to the crystallization region. The crystallization temperature of rPP was around 115 °C, but there were two peaks, which might be caused by the complex source and composition of rPP. It can also be seen from Figure 7 that the melting temperature of rPE was around 123 °C and a wide peak also appeared at around 109 °C, indicating the multiplicity of the rPE structure and the relaxation peak at the edge of the crystal region. This result was consistent with the crystallization curve of rPE. However, a weak crystallization peak appeared near 161 °C, which might be due to the doping of trace amounts of PP in rPE. According to the DSC temperature rise melting curve of rPP, two melting peaks were observed at 129 °C and 160 °C. These variations might be attributed to differences in the structures of PPs, aligning with the findings from the crystallization curve.

**Figure 10:**
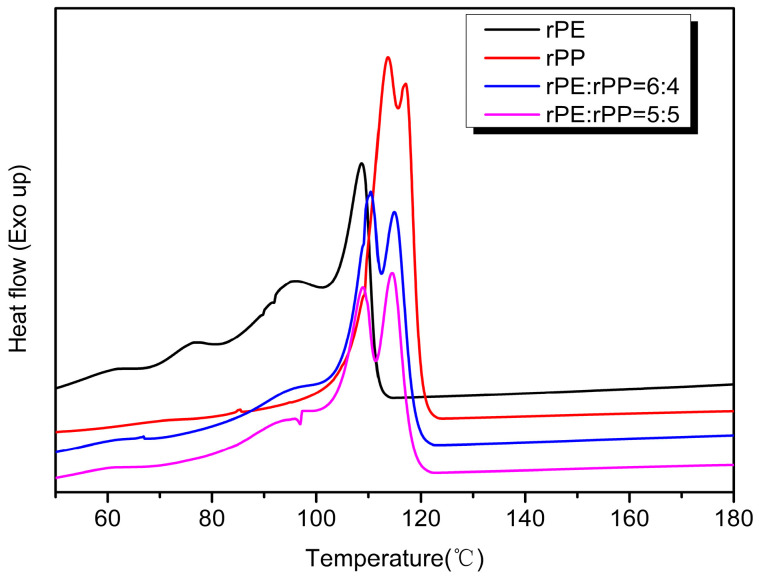
The exothermic crystallization curves of rPE, rPP, and rPE/rPP blends.

Figure 10 showed the exothermic crystallization curves of the rPE, rPP, and rPE/rPP blends. From Figure 8, the crystallization of the sample changed to some extent after rPE was blended with rPP. When the mass ratio of rPE/rPP was 6:4, the crystallization peaks of the blend were mainly located between 110 °C and 115 °C. Compared with rPE, the crystallization temperature shifted towards the high-temperature direction from 3 °C to 7 °C, indicating that the addition of rPP hindered the crystallization of rPE, increasing the crystallization temperature. Meanwhile, compared with rPE, the shoulder peaks of the blend were significantly weakened, which might be due to the addition of rPP destroying the regularity of the rPE molecular chain and reducing the crystallization ability. With the increase in rPP content, the crystallization capacity of the blend decreased further, which could be seen from the DSC curve of rPE:rPP = 5:5, and the crystallization peak area was lower than that of the rPE to rPP = 6:4 blend. The crystallization peak area of rPE/rPP was significantly reduced compared with that of rPP, proving the effect of blending on the crystallization capacity again.

**Figure 11:**
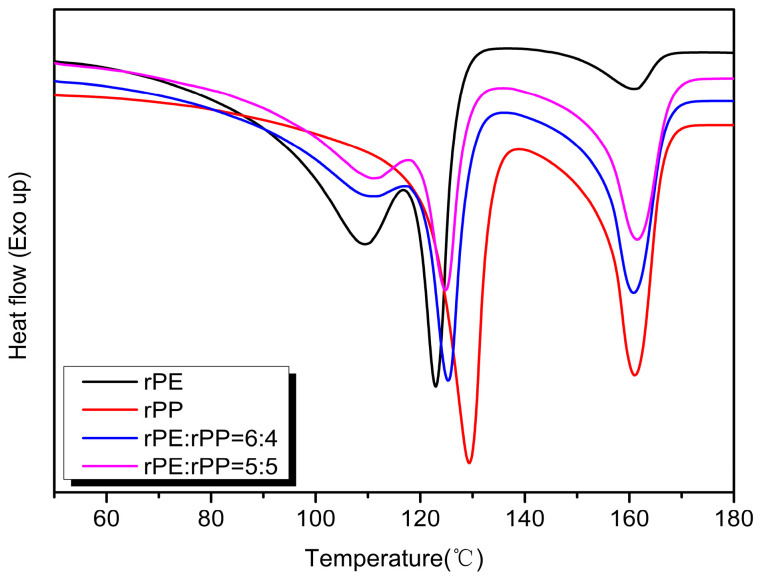
The endothermic melting curves of rPE, rPP, and rPE/rPP blends.

The endothermic melting curves of the rPE, rPP, and rPE/rPP blends were shown in Figure 11. With the addition of rPE, the main melting peak temperature of rPP decreased to 160 °C, and the melting peaks of rPE and rPP overlapped at 129 °C, resulting in a melting peak of 125 °C in the low-temperature segment of the blend. With the increase in the rPP ratio, the peak areas of melting peaks decreased, indicating that rPP had a greater impact on the crystallization ability of the blends.

### 2.8. The SEM Analysis

**Figure 12:**
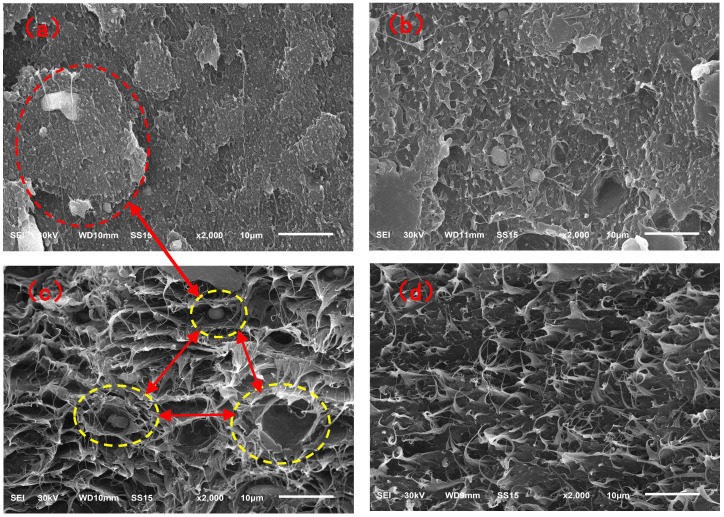
The two kinds of section micromorphology of vPE/vPP and rPE/rPP blends: (**a**) vPE/vPP = 6:4 (impact section), (**b**) rPE/rPP = 6:4 (impact sections), (**c**) vPE/vPP = 6:4 (tensile section); and (**d**) rPE/rPP = 6:4 (tensile section).

Figure 12 showed the morphology of the impact sections of the vPE/vPP and rPE/rPP blends. In Figure 12a, the SEM image of the impact section of the vPE/vPP blend revealed co-continuous structures of the two phases with relatively distinct phase boundaries and relatively flat fracture surfaces. This observation suggested a poor compatibility between vPE and vPP. When subjected to external impact, the stress transfer between the two phases was easily interrupted at the phase interface, resulting in brittle fracture. Figure 12b showed the SEM image of the impact section of the rPE/rPP blend. The boundary between the two phases appeared relatively diffuse, accompanied by numerous voids and torn fibers on the fracture surface, indicating a ductile fracture behavior in the blend. This observation suggested that the two phases exhibited a higher compatibility than the vPE/vPP blend. This enhanced compatibility might be attributed to the chemical reactions involving -OH and -COOH groups generated during the degradation of rPE and rPP. These reactions likely reduced the interfacial tension between the matrix phase and the dispersed phase, thus improving the interfacial bonding forces and leading to an increase in reactivity.

The micromorphology of the fracture surfaces after the tensile tests for the two blends was shown in Figure 12c,d. The observed features were consistent with the impact fracture morphology discussed earlier, as well as with findings from previous studies [33]. These results further indicated that the compatibility between rPE and rPP after aging had improved significantly compared to the vPE/vPP blends.

### 2.9. The Thermal Stability Analysis

**Figure 13:**
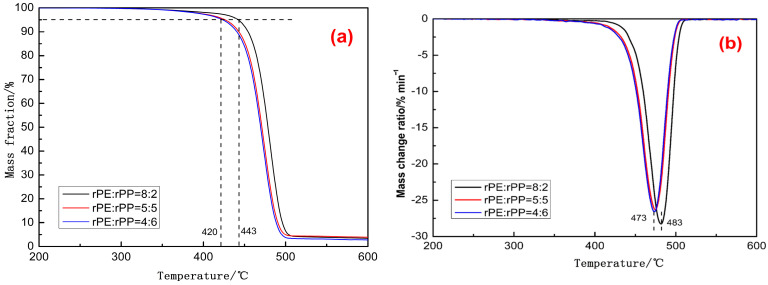
The thermogravimetry analysis of rPE/rPP with different proportions: mass fraction (**a**), mass change ration (**b**).

Figure 13 showed the thermogravimetric analysis of different ratios of rPE/rPP. The temperature of the onset of thermal degradation (T_5%_) and the temperature of the maximum thermal weight loss rate (T_max_) are usually used as parameters to measure the thermal stability of polymeric materials. When the ratio of rPE/rPP was 4/6, the T_5%_ of the sample was 420 °C, while the thermal degradation temperature range of conventional PE or PP was approximately between 300 °C and 400 °C. This indicated that a reaction might have occurred between rPE and rPP, with the increase in molecular weight, thermal degradation activation energy, and onset thermal degradation temperature; on the other hand, it might also be because rPE and rPP were in a long-term degradation process, and reactive groups, such as hydroxyl and carboxyl groups, were generated and cross-linked, raising the thermal degradation temperature. As the rPE content increased, the T_5%_ of the blends continued to rise, and, when the rPE–rPP ratio was 8/2, the T_5%_ increased to 443 °C, indicating that the contribution of rPE to the thermal stability in the blends was greater. On the one hand, this might be because rPE aged to a greater extent and cross-linking played a more decisive role in the thermal degradation experiments. On the other hand, the molecular structure of PP contained shorter methyl side chains and lacked longer side chains and aromatic ring structures. This inherent molecular configuration rendered PP more susceptible to cleavage and decomposition at high temperatures, ultimately leading to its relatively lower thermal stability. A similar pattern was evident in Figure 13b where, with an increasing proportion of rPE, there was a tendency for the T_max_ of the blend to rise. This observation confirmed the occurrence of cross-linking in rPE during prolonged degradation and suggested that some chemical reactions might have taken place between rPE and rPP, thereby enhancing the compatibility and thermal stability of the blends [34].

## 3. Experiment

### 3.1. Materials

**Figure 14:**
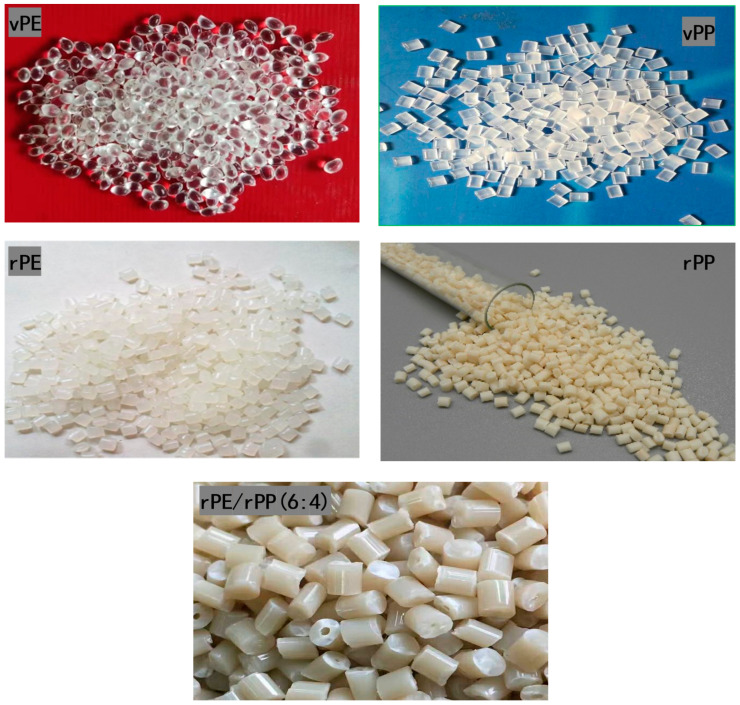
Photographs of samples obtained from blends of virgin and recycled polyethylene (vPE, rPE) and polypropylene (vPP, rPP).

The materials used in this study include virgin polyethylene (vPE), recycled polyethylene (rPE), virgin polypropylene (vPP), and recycled polypropylene (rPP) (see Table 2). Photographs of the granulates for each material are provided in Figure 14.

### 3.2. Preparation Method of Blend Samples

First, vPE, vPP, rPE, and rPP were dried in an oven at 80 °C for 2 h, and then cooled to room temperature. The blends of vPE/vPP and rPE/rPP with different proportions were premixed for 15 min at a speed of 2000 rpm by high-speed mixer (SHR-25A, Zhangjiagang City Hongji machinery Co., Ltd., Zhangjiagang, China) before being fed into the hopper of the twin-screw extruder TSE-30, Nanjing Dalit extrusion machinery Co., Ltd., Nanjing, China). Temperature controllers of the extruder (from the first zone to the ninth zone) were set from 175 °C to 190 °C, and the nose temperature was controlled at 185 °C. After cooling and air-blow-drying, the extruded strip materials were pelleted by the granulator. Then, binary blends were obtained for further test analysis.

Recycled plastics often require the incorporation of additives to enhance their performance. In this study, specific catalysts and antioxidants were used in the synthesis of virgin polypropylene (vPP) and virgin polyethylene (vPE) to modify their properties. These additives were utilized in minimal quantities. However, the rPE and rPP blends described were prepared using a twin-screw extruder without the addition of any further reagents during the preparation process. Therefore, the minimal quantities of additives used in vPP and vPE are not expected to significantly influence the properties of the rPE/rPP blends discussed in this study.

### 3.3. Performance Test and Structural Characterization

MFI was obtained by melt indexer (ZRZ1452, MTS Industrial Systems (China) Co., Ltd., Shanghai, China) according to GB/T3682-2000 (China standard, the same as later paper) [35], and the test temperature was 230 °C and the test load was 2.16 kg.

The VPE, rPE, VPP, rPP, and rPE/rPP injection molding processes involve heating sleeve temperatures in each section set at 180 °C, 185 °C, 190 °C, and 190 °C, respectively. The mechanical properties of the splines were tested (SANS CMT6104, Shenzhen SANS testing machine Co., Ltd., Shenzhen, China) using the following methods: (i) GB/T1043.1-2008 [36] test notch impact strength was conducted. A 45° notch was made on the impact spline. The stress was relieved at 25 °C for 24 h, followed by conducting the impact test at room temperature. The test instrument used was a cantilever beam impact tester with a pendulum type of 1-J; (ii) Tensile strength was tested at room temperature according to GB/T10400.1-2006 [37]. The fixture distance was set at 115 mm, and the tensile speed was 50 mm/min; (iii) Bending strength was tested according to GB/T9341-2008 [38]. The length of the sample was 120 mm, the distance of the fulcrum was 64 mm, and the bending speed was 2 mm/min. For each conducted test, a total of 5 to 6 samples were thoughtfully selected and tested, with the subsequent averaging of results to ensure the accuracy and reliability of our findings.

FTIR spectroscopy was carried out by Fourier infrared spectrometer (8400S, Shimadzu Corporation of Japan, Kyoto, Japan) to identify the probable reactions between vPE and vPP or rPE and rPP, scanning range was between 500 cm^−1^ and 4000 cm^−1^, the resolution was 4 cm^−1^, and it was performed 32 times. The FTIR spectra for the granulates were obtained after the samples had been thoroughly dried. This step was crucial to ensure that the spectra accurately reflect the inherent chemical properties of the granulates without interference from moisture. The FTIR experiments conducted in this study are primarily intended to elucidate the mixing mechanism between rPP and rPE. It is important to note that our analysis focused on a limited set of randomly selected ratios for a more controlled examination.

The enthalpy and temperature were calibrated using differential scanning calorimeter (DSC, Q100, America TA INSTRUMENTS, New Castle, DE, USA) with metal indium (In), and the flow rate of N_2_ was 50 mL/min. Firstly, the sample was heated from room temperature to 200 °C for 3 min to eliminate the thermal history of the sample. And, then, the sample was cooled from 200 °C to 40 °C for 3 min to record the cooling crystallization curve of the sample. Finally, the sample was heated from 40 °C to 200 °C to record the temperature rise melting curve of the sample, and the glass transition temperature (T_g_) of rPP and rPE was recorded. The heating and cooling rates of the samples were 20 °C/min.

During the micromorphology test, the scanning voltage was 30 kV and the scanning multiple index was 2000×. The scanning electron microscope was used for the test; the model was JSM-6510, and was purchased from Hitachi, Tokyo, Japan.

Thermal stability was analyzed by integrated thermal gravimetric analyzer (HCT-1, Beijing Hengjiu scientific instrument factory, Beijing, China); the samples with different proportions of rPE/rPP were dried at 70 °C for 12 h; and 5~10 mg samples were taken into the crucible and heated from room temperature to 800 °C under nitrogen protection at a heating rate of 10 °C/min and a nitrogen rate of 50 mL/min. TGA curves of samples were recorded and DTG curves were calculated.

## 4. Conclusions

In this paper, a range of crucial parameters including the melt index, infrared spectrum, mechanical properties, crystallization characteristics, and microscopic morphology within the contexts of vPE, rPE, vPP, rPP, and their intricate blends were comprehensively examined. Through this rigorous analysis, we arrived at the following significant conclusions:

(1) The degradation effects on rPE and rPP became evident through distinct changes in their melt index during natural usage. While the blend of vPE and vPP existed in primarily physical interactions, leading to slight increases in viscosity and minor decreases in melt index, the rPE/rPP blend displayed a marked viscosity elevation and significant melt index reduction due to interactions between the aged hydroxyl and carboxyl groups, inducing esterification reactions.
(2) Long-term natural degradation led to varied mechanical property degradation in rPE and rPP, resulting in a reduced impact strength, fracture elongation, tensile strength, and bending strength. Blending rPE and rPP yielded noteworthy changes in notch impact strength and fracture elongation, reaching peak performance at a 6:4 ratio. This improvement was attributed to esterification reactions between the aged hydroxyl and carboxyl groups, corroborated by the FTIR spectra and microscopic morphology analyses.
(3) The molecular simulation results showed that the number of hydrogen bonds in the system with an rPE–rPP ratio of 6:4 was much larger than that in the system with an rPE–rPP ratio of 8:2. The enthalpy of the former system was also much smaller than that of the latter, indicating that the system was more stable and had the best mechanical properties when the rPE–rPP ratio was 6:4.
(4) Trace amounts of PP might exist in rPE. Blending lowered the crystallization capacity of both rPE and rPP, increased rPE’s crystallization temperature, reduced rPP’s crystallization peak area, and emphasized rPP’s influence on the blend’s crystallization capacity.
(5) Thermal analysis indicated potential cross-linking and chemical compatibility between rPE and rPP in the blend, resulting in enhanced thermal stability. This outcome was supported by FTIR spectra analysis, suggesting improved blend properties through significant cross-linking and enhanced the compatibility between the components.

This study’s comprehensive investigation into the degradation effects on rPE and rPP, along with their blends, might yield crucial insights for enhancing the recycling efficiency and reducing the production costs of waste plastics. By elucidating the intricate interplay of thermo-mechanical and chemical properties, our findings offer practical strategies for sustainable plastic waste management, particularly significant for the prevalent rPE and rPP. While the study provides valuable insights into rPE/rPP blends, the conclusions are based on specific types of recycled polyethylene and polypropylene. Variations in material properties among different types of rPE and rPP may influence the performance of the blends. Further research with a broader range of materials is needed to generalize the findings, explore advanced techniques to mitigate degradation effects, and optimize the performance of recycled plastic blends.

## Figures and Tables

**Table 1:** The mechanical properties of vPE, rPE, vPE, and rPE.

Materials	Tensile Strength (MPa)	Elongation (%)	Notched Impact Strength (kJ/m^2^)	Bending Strength (MPa)
vPE	38.57	168.44	8.16	32.52
rPE	24.12	44.23	4.05	22.63
vPP	31.26	107.45	7.18	29.43
rPP	27.90	67.52	5.19	21.43

**Table 2:** Information of raw material.

Materials	Sources	Model	Manufacturers	City/Country
vPE	Business mailing sample	220J	Sinopec Yangzi Petrochemical Co., Ltd.	Nanjing/China
rPE	Medical PE plastic bottle	R−220J	Dongguan Zhongmin New Material Technology Co., Ltd.	Dongguan/China
vPP	Business mailing sample	1016	Liaoyang Petroleum Chemical Fiber Company	Liaoyang/China
rPP	Electrical enclosure	R-1016	Shanghai Jinfa Technology Development Co., Ltd.	Shanghai/China

## Data Availability

The research data are available by contacting the corresponding author.

## Supplementary Materials

The following supporting information can be downloaded at: https://www.mdpi.com/article/10.3390/molecules29184499/s1, Video S1: MD Simulation Trajectory.
